# Oxytocin and vasopressin: linking pituitary neuropeptides and their receptors to social neurocircuits

**DOI:** 10.3389/fnins.2015.00335

**Published:** 2015-09-24

**Authors:** Danielle A. Baribeau, Evdokia Anagnostou

**Affiliations:** ^1^Department of Psychiatry, University of TorontoToronto, ON, Canada; ^2^Autism Research Centre, Bloorview Research Institute, Holland Bloorview Kids Rehabilitation HospitalToronto, ON, Canada

**Keywords:** oxytocin, vasopressin, vasopressin receptor subtype 1a, OXTR, autism

## Abstract

Oxytocin and vasopressin are pituitary neuropeptides that have been shown to affect social processes in mammals. There is growing interest in these molecules and their receptors as potential precipitants of, and/or treatments for, social deficits in neurodevelopmental disorders, including autism spectrum disorder. Numerous behavioral-genetic studies suggest that there is an association between these peptides and individual social abilities; however, an explanatory model that links hormonal activity at the receptor level to complex human behavior remains elusive. The following review summarizes the known associations between the oxytocin and vasopressin neuropeptide systems and social neurocircuits in the brain. Following a micro- to macro- level trajectory, current literature on the synthesis and secretion of these peptides, and the structure, function and distribution of their respective receptors is first surveyed. Next, current models regarding the mechanism of action of these peptides on microcircuitry and other neurotransmitter systems are discussed. Functional neuroimaging evidence on the acute effects of exogenous administration of these peptides on brain activity is then reviewed. Overall, a model in which the local neuromodulatory effects of pituitary neuropeptides on brainstem and basal forebrain regions strengthen signaling within social neurocircuits proves appealing. However, these findings are derived from animal models; more research is needed to clarify the relevance of these mechanisms to human behavior and treatment of social deficits in neuropsychiatric disorders.

## Introduction

Oxytocin and arginine vasopressin (AVP) are neuropeptides synthesized in the hypothalamus and secreted from the posterior pituitary gland. Oxytocin was first described for its important role in stimulating uterine contractions and milk let down after birth, while AVP is central to water homeostasis by regulating urine concentration at the level of the kidney. In addition to these physiologic functions, both peptides are now understood to mediate numerous social behaviors in mammals.

**Figure 1:**
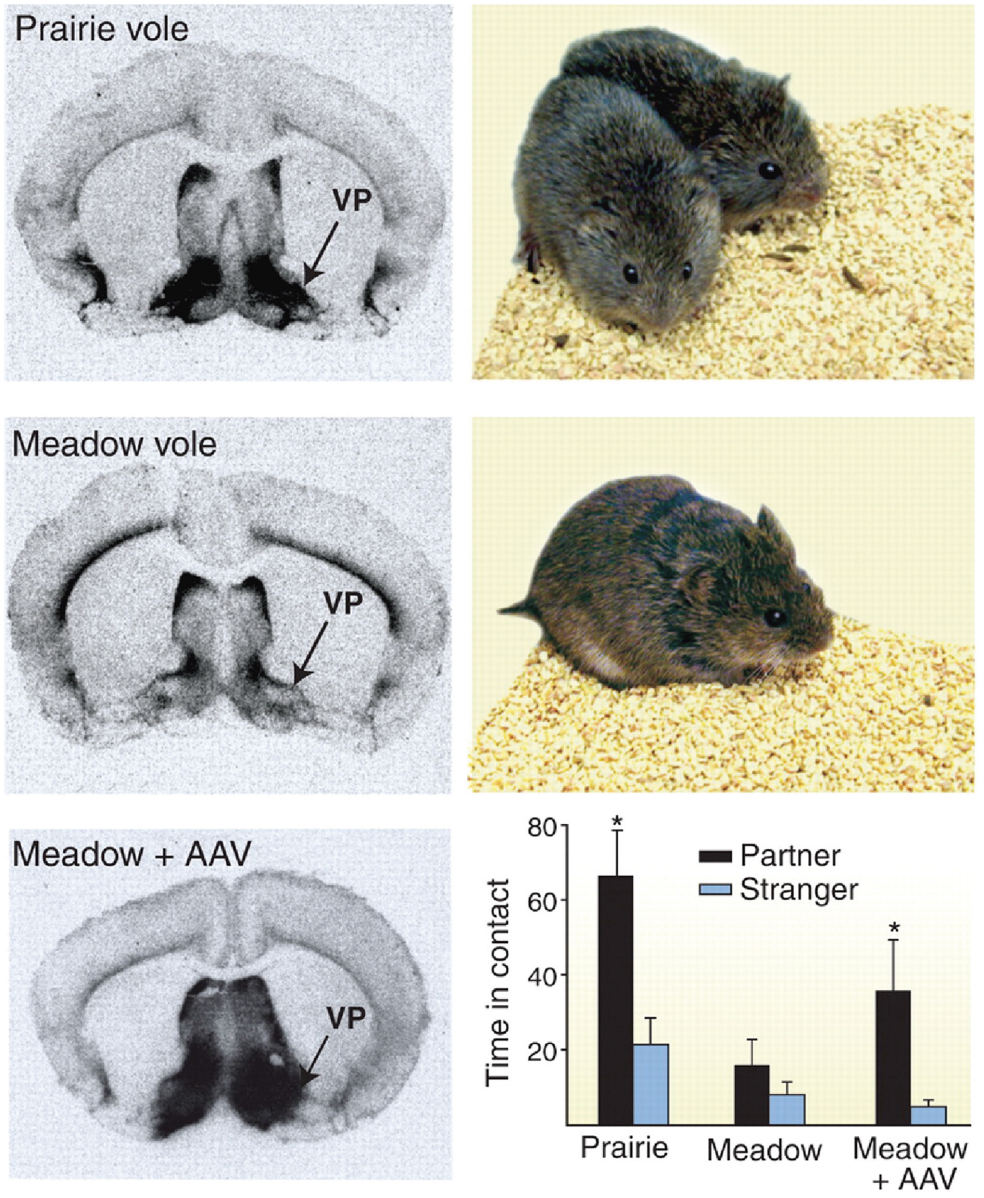
**Example of receptor autoradiography study in voles showing higher density of vasopressin receptor 1a staining in monogamous prairie voles (top) as compared to polygamous meadow voles (middle)**. When receptor expression was increased using an adeno associated viral (AAV) vector, polygamous meadow voles demonstrated more preferential contact with their partners **(bottom)**. Figure reproduced with permission from *Science*, (Donaldson and Young, 2008) adapted from research presented by Lim et al. (2004). ^*^p < 0.05.

The role of the oxytocin and vasopressin systems in social functioning has developed out of a large body of animal research, focusing primarily on rodents. This literature has been extensively reviewed elsewhere (Wang et al., 1998; Insel, 2010). For example, oxytocin has been shown to be an important regulator of maternal behavior in female rats, with central injection of this molecule triggering protective and nursing behavior toward pups. Similarly, both peptides mediate affiliative behavior in prairie voles, although effects differ by sex. In male prairie voles, for example, manipulation of this system with AVP receptor antagonists attenuates preferential association with a partner after mating, while central administration of this peptide triggers pair bonding even in the absence of mating behavior (see Figure 1) (Wang et al., 1998; Cho et al., 1999). Accordingly, there is growing interest in oxytocin and vasopressin as modulators of social behavior and functioning in humans; either as a potential explanatory factor for social differences in typically developing individuals, or as a possible precipitant of and/or treatment for social deficits in neurodevelopmental disorders such as autism spectrum disorder (ASD).

Indeed, there is ample research to suggest to that common genetic variation in the receptor structure for these molecules may impact on some aspects of social functioning in humans; additionally, central administration of oxytocin seems to encourage certain social behaviors and cognitive capacities (Meyer-Lindenberg et al., 2011). Functional neuroimaging studies further support a link between these neuropeptides and activity in specific brain regions implicated in social communication and behavior. It remains unclear whether disruption of the oxytocin/vasopressin system contributes to the etiopathogenesis of ASD, however. A single case report describes a family in which a rare mutation in the oxytocin receptor was detected in an individual with ASD (Gregory et al., 2009), and a recent meta-analysis suggests certain common genetic variants may be over represented in autism (Loparo and Waldman, 2014). However, new research has shown that both peripheral oxytocin levels, and common genetic variation in the oxytocin receptor affect social communication abilities in family members of individuals with ASD as well, irrespective of diagnosis (Skuse et al., 2014; Parker et al., 2014). Accordingly, dozens of trials in which oxytocin or vasopressin are manipulated with pharmacotherapy are underway, showing early evidence as a potential treatment for social deficits (reviewed by Baribeau and Anagnostou, 2014). Despite this growing interest, a model that links the molecular and cellular activity of these peptides to social neurocircuits detectable on neuroimaging remains elusive.

Accordingly, the following review intends to summarize the current literature with respect to underlying mechanisms via which neuropeptides affect social processes in humans, focusing on the oxytocin and vasopressin systems. We aim to provide a sequenced narrative review of research evidence, following a micro- to macro- level trajectory. Specifically, we begin by summarizing what is known about the synthesis and secretion of these peptides, followed by a discussion on the distribution, structure, and activity of their respective receptors. Next, current models associating these peptides to specific effects on neurons, neurotransmitters, and microcircuits will be reviewed. We will then correlate this research with current functional neuroimaging literature examining responses to experimental manipulation of these systems. Potential implications for, and associations with ASD are included throughout. The aim is to provide a non-technical overview of this field, to synthesize results from overlapping yet distinct areas of science, and to identify knowledge gaps in need of further exploration.

## Oxytocin and vasopressin molecules: synthesis and release

Oxytocin and vasopressin are related pituitary non-apeptides; they consist of nine amino acids in a cyclic structure. These molecules differ by only two amino acids, at position 3 and 8 (isoleucine and leucine in oxytocin are replaced by phenylanine and arginine in vasopressin, respectively). Related peptides are detectable in all vertebrate species and are thought to have evolved from similar parent compounds. Both oxytocin and vasopressin are coded in a precursor form on chromosome 20 (Gimpl and Fahrenholz, 2001).

**Figure 2:**
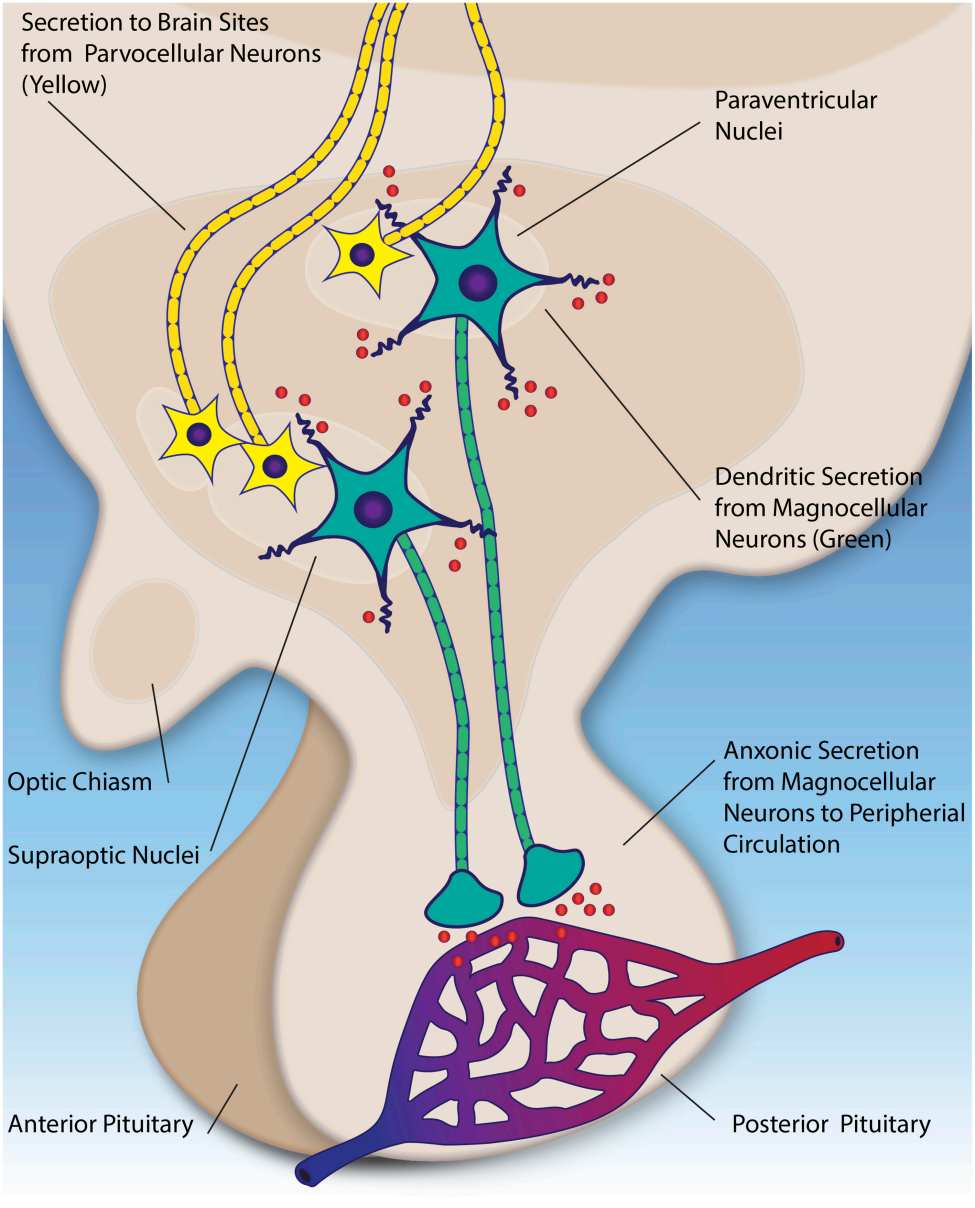
**Parvocellular neurons (yellow) secrete oxytocin and vasopressin (red) to numerous brain regions, including the amygdala, brainstem, and anterior pituitary**. Magnocellular neurons (green) in the hypothalamic nuclei secrete oxytocin and vasopressin into the peripheral circulation via the posterior pituitary (axonic secretion). Additionally, they secrete these peptides into the extracellular fluid the hypothalamus (dendritic secretion).

Both molecules are synthesized in overlapping regions of the hypothalamus, primarily in large magnocellular neurons situated in the supraoptic and paraventricular nuclei. These neurons project their axons to the posterior pituitary, where the peptides are stored in vesicles until action potentials trigger their release into the peripheral circulation (for example during labor, or imbalance of water homeostasis) (Ludwig and Leng, 2006) (see Figure 2). Oxytocin and vasopressin molecules that have been released in this way, through the axon projections, are for the most part prevented from re-entering the central nervous system (CNS) via the blood brain barrier; however, very small amounts of peripherally administered peptides (e.g., < 1%) do appear to cross over into the cerebral spinal fluid (CSF) (Mens et al., 1983; Opacka-Juffry and Mohiyeddini, 2012). It has been shown that oxytocin and vasopressin concentrations can be up to 1000X higher in the brain than the peripheral blood, indicative of a potentially important role for both molecules in the central nervous system (CNS) (Ludwig and Leng, 2006). While earlier studies suggested potentially lower oxytocin and vasopressin levels in the plasma of children with ASD as compared to typical children (Modahl et al., 1998; Al-Ayadhi, 2005), subsequent research has shown that plasma oxytocin levels tend to be similar within members of the same family, irrespective of a diagnosis of autism, although do correlate with social communication abilities overall (Parker et al., 2014). Of note, the methodology used to quantify plasma oxytocin levels in humans has varied across studies, which may have affected the reliability of results (Szeto et al., 2011).

Oxytocin has a single receptor (OXTR) encoded on chromosome 3, whereas vasopressin has three types of receptors, AVPR1a and AVPR1b (also called V3) and V2, on chromosome 20 (De Keyzer et al., 1994; Thibonnier et al., 2002). AVPR1a is present primarily on vascular smooth muscle, in the liver, and on neurons; AVPR1b/V3 is detectable in the anterior pituitary; and the V2 receptor is found primarily in the kidneys. Information on the central distribution, structure, and function of these receptors will be discussed further in subsequent sections. Outside of the brain, oxytocin receptors are detectable in humans in high concentrations in the uterus, gradually increasing in number over the course of pregnancy. Tissue taken from hysterectomy or cesarean section at different gestational time points in pregnant women has shown a significant and rapid up regulation (e.g., 200-fold) of oxytocin receptor expression around the onset of labor, facilitating uterine contractions (Fuchs et al., 1984). Many other tissues and organs, including ovaries, testis, mammary glands, kidneys, thymus, pancreas, adrenal, and even adipose tissue, have been shown to express oxytocin and/or vasopressin receptors in different species; some studies even suggest exogenous synthesis of oxytocin can take place at certain peripheral sites (see Gimpl and Fahrenholz, 2001 for a detailed review). As well, both oxytocin and vasopressin can exert effects on the cardiovascular system by affecting blood pressure, vasodilation, diuresis, and water intake (Pittman et al., 1982; Petersson et al., 1996). This finding may be of relevance in ASD given emerging evidence of aberrant autonomic functioning and heart rate reactivity in this condition (Ming et al., 2005; Kushki et al., 2013).

The synthesis and release of oxytocin and vasopressin in the CNS is of primary importance for models associating these peptides with social behavior. Our knowledge of these processes stems almost exclusively from research in animal models, primarily in rodents (see Ludwig and Leng, 2006, for an extensive review). Central release appears to be mediated through two pathways, distinct from the peripheral secretion described above (see Figure 2). First, both peptides are produced in in hypothalamic neurosecrotor neurons (parvocellular neurons), whose axons project to the anterior pituitary and other brain regions in rodents (Castel and Morris, 1988; Ludwig and Leng, 2006). Parallel to this, the magnocellular neurons (mentioned in the previous section) also have been shown to secrete oxytocin and vasopressin from their dendrites (as opposed to the axons) in the hypothalamus (Pow and Morris, 1989). This mechanism appears to be separate and distinct from the axonal secretion in the posterior pituitary, and potentially contributes feedback to the overall system (Ludwig et al., 2002). Following secretion, these peptides are thought diffuse throughout the extracellular space, serving a neuromodulatory effect on surrounding brain tissue (Landgraf and Neumann, 2004).

Across histological studies, many investigators have highlighted extensive axonic and dendritic projections extending from the oxytocin and vasopressin neurons in the hypothalamus. In mice, for example, dendrites arising from hypothalamic magnocellular neurons were shown to display a corkscrew morphology, projecting posteriorly toward the third ventricle, and also extending beneath the pia layer of the base of the brain (Castel and Morris, 1988). Similarly, oxytocin neurons in the paraventricular nucleus of the hypothalamus have been shown to project axons long distances across the basal forebrain in rats, with extensive branching and three dimensional orientations extending potentially as far as the nucleus accumbens, amygdala, hippocampus, and into the somatosensory cortex (Knobloch et al., 2012; Grinevich et al., 2015). Interestingly, these axonal projections are only detected in adult animals, and are absent in prenatal and early postnatal studies. Dendritic secretion has been shown to be of central importance in animal models of social stress (Ludwig and Leng, 2006), while axonic secretion has been shown to effect fear responses in mice (Knobloch et al., 2012). As summarized in Table 1, oxytocin receptors have been detected on cell fibers in the hypothalamus, brainstem, and throughout the limbic system in human brains (Boccia et al., 2013).

**Table 1:** **Distribution of oxytocin receptors in the central nervous system**.

	**Non-human Primates**				**Humans**	
References	Boccia et al., 2001	Boccia et al., 2007	Freeman et al., 2014a	Freeman et al., 2014b	Loup et al., 1989, 1991	Boccia et al., 2013
Species	Macaque	Macaque	Macaque	Titimonkey	Human	Human
Method (Right)	Monoclonal antibody	Oxytocin antagonist	Autoradiography	Autoradiography, mRNA	Autoradiography	IHC
Brain regions (Below)						
**CORTICAL AREAS**						
Frontal cortex		+			−	±
Temporal cortex					−	−/−
Parietal cortex		−				−/−
Cerebellar cortex		−			−	
Occipital cortex		−		++		
Retrosplenial cortex/Subcallosal area						+/+
Cingulate cortex						+++/+
**BASAL FOREBRAIN**						
Diagonal band of Broca					+++	+++
Basal nucleus of Meynert			+++	++	+++	
Septal nuclei	++	+++			+++	+++/+++
**BASAL GANGLIA**						
Caudate nuclei		−			−	
Globus pallidus					+	
Nucleus accumbens				−	−	−/−
Putamen					−	
**LIMBIC SYSTEM**						
Amygdala		+		−	−	+++/+++
Hippocampus		++			−	−/−
Parahippocampus/Hippocampal formation				+++	−	+++/+++
Olfactory system					−	+/+
Thalamus				++		
**HYPOTHALAMUS**						
Anterior Hypothalamus	++	+++			++	++/++
Posterior Hypothalamus					++	+++/+++
Tuberal Hypothalamus			+++		+	++/−
**BRAIN STEM**						
Midbrain		−	+++		++	−/−
Pons		−	+++	++	+	−/−
Medulla		−		++	++	+++/+++

Cell bodies/Cell fibers. IHC, immunohistochemistry; +++, high density binding; ++, moderate density binding; +, low density binding; −, no binding. Boxes left blank not explicitly described in manuscript. Note that tracts and nuclei have been grouped by brain region. See respective reference for more details.

In animal models, the central vs. peripheral secretion of these peptides have been shown to follow distinct timelines in their responses, with brain levels peaking later, and lasting for longer than elevations in blood levels. For example, injection of a hypertonic solution into the peripheral circulation of rats triggered elevations in peptide levels that peaked at 150 min in the CNS as opposed to 30 min in the periphery (Ludwig et al., 1994). When either oxytocin or vasopressin was injected into the rat peripheral circulation, central elevations in the CSF were also shown to persist for much longer, and were cleared more slowly than peripheral levels (Mens et al., 1983). In order to deliver oxytocin directly to the central nervous system for therapeutic uses in ASD, intranasal sprays have been developed which show rapid rises in CSF oxytocin levels within 10 min of application in macaques (Dal Monte et al., 2014). In humans, single intranasal administration of oxytocin led to elevated plasma levels for approximately 90 min afterwards (Gossen et al., 2012). In primates, levels of both oxytocin and vasopressin have been shown to fluctuate over the course of the day, with different patterns in the plasma vs. the CSF; specifically, CSF levels tended to correspond with periods of daylight, while plasma levels did not (Perlow et al., 1982).

Interestingly, oxytocin and vasopressin neurons have receptors for their own secreted neuropeptides on their cell surfaces, and are able modulate their own release, respectively, without necessarily triggering action potentials (Gouzenes et al., 1998; Ludwig et al., 2002). Evidence derived from mouse models indicates that a transmembrane glycoprotein called CD38 must be present to facilitate depolarization-induced oxytocin secretion in the pituitary, and that blockade of this molecule interrupts mouse maternal and social behavior (Jin et al., 2007; Lopatina et al., 2012). Specific common genetic variants in CD38, and reduced CD38 expression on lymphoblastoid cells, have been associated with ASD (Higashida et al., 2010; Lerer et al., 2010). Once released via either central pathway, the peptides then diffuse throughout the extracellular space, and can be detected across the brain, where they act on their respective receptors (Ludwig and Leng, 2006). The mechanism of action of peptide binding and central distribution of these receptors will be discussed in the next sections.

## Neuropeptide receptors: structure and function

Both oxytocin and vasopressin receptors are G-protein coupled, each with seven transmembrane alpha-helices connected via extra and intracellular loops (Kimura et al., 1992) (for a detailed review of the receptor structure see (Zingg and Laporte, 2003) or Gimpl and Fahrenholz, 2001). There is cross reactivity in binding of each peptide with its respective receptor; oxytocin binds to the oxytocin receptor with only 10x greater affinity than vasopressin, for example (Kimura et al., 1994). The strength of binding of the neuropeptide into its specific binding pocket can be manipulated by mutational analyses; for example, single amino acid substitutions at key structural areas can significantly reduce or eliminate peptide binding (Hausmann et al., 1996; Postina et al., 1996). A natural example of this occurs in nephrogenic diabetes insipidis, where a point mutation affecting arginine disrupts the V2 receptor structure, resulting in an inability to concentrate urine in affected individuals (Bichet et al., 1993; Birnbaumer et al., 1994; Rosenthal et al., 1994). The genetic sequences coding for mouse, rat, and human oxytocin/vasopressin receptor genes are conserved across species, with over 80% identical amino acid residues. There are subtle differences in receptor function across species, however. For example, in mice and rats, there are two N-glycosylation sites in the extracellular NH2 region, while in humans and primates, there appear to be three (Gimpl and Fahrenholz, 2001). *In vitro* studies suggest that the binding affinity of endogenous oxytocin for its receptor is comparable in humans, rats, and mice. However, there is significant variability the affinity constants of the synthetic oxytocin analog TGOT for OXTR across different species (Busnelli et al., 2013). As such, animal models using synthetic OXTR agonists may not necessarily reflect human physiology. Although numerous studies have associated common genetic variation in the human oxytocin receptor with ASD or social deficits (Bakermans-Kranenburg and van Ijzendoorn, 2014; Loparo and Waldman, 2014), the impact of these genetic changes on the structure and function of the oxytocin receptor *in vivo* is unclear at the present time.

The neuropeptide binding to its respective receptor triggers a conformational change in the receptor structure, leading to downstream activation of G proteins, and subsequent Ca^2+^ release from intracellular stores. Potential downstream effects include phosphorylation of intracellular proteins, activation of nitric oxide synthase leading to vasodilation, smooth muscle contraction, gene transcription, and increased excitability of neurons. The specific effects of receptor activation seem to vary by organ and tissue (Zingg and Laporte, 2003). For both OXTR and V2, there is evidence for rapid receptor desensitization, via receptor internalization. *In vitro* studies suggest this desensitization effect may be present minutes to hours after exposure to the peptide, and can result in >50% internalization of receptors (Gimpl and Fahrenholz, 2001). Internalized receptors are not degraded, however; approximately 85% of receptors return to the cell surface within 4 h (Conti et al., 2009). This internalization process may impact on social functioning. In mice, for example, chronic twice-daily administration of intranasal oxytocin reduced oxytocin receptor expression in the brain, and decreased some social behavior, while acute administration increased social behaviors, although findings varied by dose (Huang et al., 2014). The mechanism driving receptor internalization appears to involve receptor congregation with beta-arrestin into clathrin-coated pits (Oakley et al., 2001). Both oxytocin and vasopressin receptors show capacity to form hetero- homo- or oligo-dimers *in vitro*; it is unclear to what extent formation of receptor complexes is a biologically important process *in vivo* (Cottet et al., 2010).

## Mediators of peptide and receptor transcription, synthesis, and secretion

Several mediators of transcription, synthesis, and secretion/expression of the oxytocin and vasopressin peptides and their respective receptors have been described in various species (Burbach et al., 1995; Jorgensen et al., 2002; Weiser et al., 2008). Specifically, activation of transcriptional promoters upstream of the oxytocin or vasopressin genes via estrogen receptor binding, thyroid hormone receptor binding or retinoic acid receptor binding has been shown *in vitro* (Richard and Zingg, 1990, 1991; Shapiro et al., 2000; Pak et al., 2007). Sex steroids, including estrogen, progesterone, and testosterone, and pro-inflammatory cytokines, such as interleukin-6 and interleukin 1-beta, have been shown to impact on OXTR expression levels in various tissues in animal models (Kimura et al., 2003). Of note, in ASD, abnormal levels of inflammatory cytokines have been described (Croonenberghs et al., 2002). Various neurotransmitters, including noradrenaline and serotonin have also been shown to play a role in modulating neuropeptide secretion in both the central and peripheral circulation (Vacher et al., 2002). Restraint of a rat induced elevation in oxytocin and vasopressin levels, which could be inhibited by blocking specific serotonin receptors, for example (Jorgensen et al., 2002). The discussion on the relationship between sex steroids, neurotransmitters, and the behavioral effects of oxytocin and vasopressin is elaborated in Section Association with Neurotransmitters and Social Circuits.

## Receptor distribution in the central nervous system

The specific distribution of oxytocin and vasopressin receptors in the human brain has been difficult to study precisely. Unlike other neurotransmitter systems, a positron emission tomography (PET) radioligand has yet to be identified with adequate receptor specificity and CNS penetration for use in humans. Early trials testing tentative oxytocin PET ligands are currently underway in animal models, however (Smith et al., 2013a,b).

Accordingly, investigators have relied on post-mortem tissue analysis via receptor autoradiography and immunohistochemistry (IHC) in small samples of human subjects; alternatively, inferences can be drawn from data derived using similar techniques in animal studies. Both approaches have associated limitations. For example, certain oxytocin autoradiographic receptor ligands have been shown to have significant cross reactivity with AVP receptors (Toloczko et al., 1997). Acquisition of post-mortem brain tissue for analysis can prove challenging. Only typical adult brains have been studied so far. Additionally, receptor distribution in animal models has been shown to vary significantly depending on the age of the animal (Tribollet et al., 1989), and the species studied (Raggenbass et al., 1989; Gimpl and Fahrenholz, 2001). Translating information on receptor distribution in rodents to humans is particularly problematic, as patterns vary profoundly even between related rodents species.

Autoradiography uses a radioactive ligand tracer applied to mounted tissue sections and analyzed under a microscope, circumventing any difficulties with receptor penetration of the blood brain barrier. Tribollet and colleagues were some of the first investigators to apply this approach to the rat brain (Tribollet et al., 1988), suggesting that AVP and OT receptor distributions were sufficiently distinct. AVP receptors were detected primarily in the limbic system and hypothalamus; oxytocin receptors were also detected in the hypothalamus, as well in the olfactory tubercle and hippocampus. The relevance and translation of this information to the human brain was unclear at the time.

Loup and colleagues subsequently applied autoradiography to study oxytocin receptor distributions in 12 post-mortem human brains in typical adults, free of psychiatric illness (Loup et al., 1989). They used [^3^H]OT^9−11^ and a newly synthesized OXTR ligand [^125^I]OTA, applied to tissue sections. In their first publication on receptor distribution in the brain stem and spinal cord, they identified oxytocin binding in numerous overlapping tracts involved in sensory, motor, and autonomic function (e.g., the substantia nigra, the substantiae gelatinosa of the spinal trigeminal nucleus, the dorsal horn of the upper spinal cord, as well as the nucleus of the solitary tract) (Loup et al., 1989).

The same investigators subsequently applied this technique to the entire brain, while adding an AVP receptor ligand ([^3^H]AVP) to distinguish AVP binding from binding to the oxytocin receptor, in both cortical and subcortical regions. Oxytocin and AVPR1a binding was detected in numerous limbic and autonomic pathways, with some distinct areas and some overlapping. In the cortical sections, oxytocin binding was strongest in the basal forebrain and nearby structures, including (1) specific cholinergic tracts (i.e., the basal nucleus of Meynert and the nucleus of the vertical limb of the diagonal band of Broca); (2) in the ventral part of the lateral septal nucleus (which relays between the hippocampus, thalamus, and midbrain); and (3) in parts of the hypothalamus and basal ganglia. Importantly, no OXTR binding was identified in the nucleus accumbens, caudate, putamen, hippocampus, amygdala, or in the frontal, temporal, or cerebellar cortices (see Table 1). The areas with strongest AVP binding were non-overlapping as compared to oxytocin, in the dorsal part of the lateral septal nucleus, and in certain thalamic nuclei as well (Loup et al., 1991). Many other areas showed weaker binding for AVP, including the hippocampal formation, parts of the basal ganglia, and specific brainstem nuclei (e.g., nucleus of the solitary tract and spinal trigeminal nucleus). However, the ligand used to test for oxytocin receptor binding in these studies (^125^I-OTA), was subsequently shown to also bind AVPR1a receptors with equal strength as to the oxytocin receptor, calling into question the reliability of previous findings (Toloczko et al., 1997).

Recent literature is limited with respect to more concise localization of oxytocin and vasopressin receptors in the central nervous system in humans (see Table 1). One study in human brains used immunohistochemistry with a monoclonal antibody targeted to the oxytocin receptor (Boccia et al., 2013). As with earlier work, oxytocin receptors were identified in the hypothalamic and limbic areas, including the vertical limb of the diagonal band. The authors specifically commented on a lack of oxytocin receptor detection in the raphe nucleus of the brainstem. Unlike in previous human autoradiographic studies, however, oxytocin receptors were also detected in the anterior cingulate, amygdala, and in the olfactory nucleus. Of note, IHC staining of OXTR receptors was detected on both the cell membrane and in the cytoplasm of the cell body. The authors of this paper and of others (e.g., Yoshida et al., 2009) have described difficulty with reliability using immunostaining for the OXTR receptor, however, with variable results with each lot of antiserum.

Recent research in non-human primate brains may help clarify potential inconsistencies in the limited literature on human subjects. For example, Freeman et al., applied novel autoradiographic ligands for both OXTR and AVPR1a to coppery titi monkey brains, a socially monogamous species (Freeman et al., 2014b). They found AVPR1a receptors diffusely throughout the brain, with oxytocin receptors more localized to specific areas (e.g., the hippocampus and surrounding areas, nucleus basalis, thalamus, visual cortex, and brainstem structures). They confirmed their findings regarding OXTR by measuring mRNA expression levels, which overlapped with autoradiographic binding for OXTR (Freeman et al., 2014b). Similarly, Freeman et al., applied the same technique to macaque brains, and again detected more diffuse AVPR1a binding, with more localized OXTR binding. In the macaques, regions where OXTR bound most strongly involved sensory processing of visual and auditory stimuli, (e.g., nucleus basalis, pedunculopontine tegmental nucleus, superior colliculus, trapezoid body in the brainstem, hypothalamus) and seemed to overlap with many cholinergic pathways of the basal forebrain (Freeman et al., 2014a).

Overall, limited human data in control subjects only, inconsistencies and criticisms regarding methodology, and lack of a specific PET ligand for either receptor, highlight a need for further investigation into the distribution of these receptors in the CNS. However, by looking across existing human studies, and extrapolating from primate data, several conclusions can be drawn: (1) Oxytocin and vasopressin receptors are consistently detected in the hypothalamus. (2) AVPR1a expression appears to occur more diffusely throughout the central nervous system, while oxytocin receptor expression appears more localized. (3) Oxytocin receptors have been inconsistently identified in the limbic system, with conflicting evidence regarding the amygdala. (4) Oxytocin receptor staining occurs most prominently in the basal forebrain, in certain cholinergic tracts (e.g., nucleus basalis, diagonal band of Broca) and specific brainstem nuclei (e.g., the pedunculopontine tegmental nucleus). The basal forebrain consists of a group of structures situated anterior and inferior to the striatum, including the nucleus basalis of Meynert, the diagonal band of Broca and the medial septal nuclei. It provides extensive cholinergic input to all layers of the cortex, and receives input from prefrontal regions, the nucleus accumbens and the ventral tegmental area. GABAergic basal forebrain projections to the amygdala have also been shown to modulate inhibitory signals in this region (McDonald et al., 2011). The basal forebrain is thought to play an important role in visual attention, memory, and learning, and undergoes degeneration in conditions such as Alzheimer's dementia. Future studies examining differences in the expression and distribution of these receptors in neurodevelopmental and neuropsychiatric disorders will be of particular interest moving forward.

## Association with neurotransmitters and social circuits

The complex relationships between oxytocin, vasopressin, and monoamine neurotransmitter systems have been studied in various animal models. The translation of this information to human social networks remains speculative. Below, we discuss the relationship between oxytocin, vasopressin, and various neurotransmitter systems and brain circuits.

### Serotonin

As mentioned in previous sections, serotonin activity may also contribute to the effects of oxytocin and vasopressin on social functioning, either by modulating peptide secretion, or for downstream effects on fear responses and anxiety. For example, functional activation of specific serotonin receptor subtypes was necessary to facilitate elevations in oxytocin and vasopressin levels in response to stress in rodents (Jorgensen et al., 2002). Likewise, application of serotonin to tissue sections from the hypothalamus/pituitary of the rat brain increased oxytocin and vasopressin secretion (Galfi et al., 2005). Data in rodent models suggest that the aggressive behavior stimulated as a result of AVP administration can be blocked via serotonergic activity (Delville et al., 1996a; Ferris, 1996). Oxytocin receptors are expressed on the neurons of the serotonin raphe nuclei in rats; infusion of oxytocin facilitated serotonin release from these cells and had an anxiolytic effect on rat behavior (Yoshida et al., 2009). However, a recent study in which oxytocin receptors were knocked out of the raphe nuclei in mice found deficits only in males with respect to aggression; all other social and parenting behaviors remained intact (Pagani et al., 2015). In macaques, serotonin transporters co-localized to the regions of the hypothalamus expressing oxytocin receptors (Emiliano et al., 2007). In human subjects with personality disorders, CSF levels of AVP correlated with aggression, while one of two serotonin proxy-measures was inversely associated with aggressive behavior (Coccaro et al., 1998). In children with ASD, oxytocin and serotonin plasma levels were inversely correlated with each other in one study (Hammock et al., 2012). In adults with ASD, serotonin transporter binding was lower throughout the brain on PET (Nakamura et al., 2010). Overall, animal studies suggest that serotonin receptor activation can trigger, and may be necessary to facilitate oxytocin and vasopressin secretion, while oxytocin may also stimulate serotonin release. Simultaneously, serotonin and vasopressin may have opposing effects with respect to aggressive behavior.

### Hypothalamic pituitary axis

Other investigators have shown that oxytocin and vasopressin may affect behavior by regulating stress responses through the HPA axis (Neumann, 2002). For example, rats put under a forced swim test showed central elevation of both oxytocin and vasopressin. Peripheral blood levels of oxytocin but not vasopressin, increased as well (Wotjak et al., 1998). Rats exposed to a noise stress had a dose dependent reduction in corticosteroid levels when treated with centrally administered oxytocin. Anxious behavior when exploring an unfamiliar maze was also less (Windle et al., 1997). In response to restraint, oxytocin but not vasopressin administration reduced adrenocorticotropic hormone (ACTH) and cortisol levels in rats (Windle et al., 2004). This effect resulted from reduced neuronal activity in the hypothalamus, hippocampus, and ventrolateral septum, as indicated by absence of elevations c-fos mRNA expression in these regions in the rats who were restrained and treated with oxytocin; no such effect was seen with vasopressin. In squirrel monkeys, chronic oxytocin administration reduced ACTH, but not cortisol secretion in response to social isolation (Parker et al., 2005). In humans, oxytocin administration enhanced the stress buffering effect of social support in response to a social stress paradigm, as indicated by increased calmness and reduced salivary cortisol levels in participants (Heinrichs et al., 2004). Similarly, oxytocin enhanced positive communication and reduced salivary cortisol levels during couple conflicts (Ditzen et al., 2009).

Vasopressin has been shown in animals to enhance corticotrophin releasing factor (CRF) mediated elevations in ACTH (Rivier and Vale, 1983). This process appears to be mediated via the AVPR1b receptor (Stevenson and Caldwell, 2012). It may be that oxytocin attenuates the stress response, while vasopressin might facilitate it (Bisagno and Cadet, 2014). In a rodent model, each peptide was shown to activate a different set of neurons in the amygdala, having opposite regulatory effects on excitatory input (Huber et al., 2005; Viviani et al., 2011). Interest in using AVPR1b antagonism to treat anxiety disorders has been investigated showing potential benefits in rodent models (Iijima et al., 2014), while commercial human studies are underway for AVPR1a receptor antagonists as a potential treatment for ASD (e.g., RG7314, clinical trials.gov ID: NCT01793441).

### Sex hormones

Sex hormones have been shown to be of particular importance to the central neuropeptide effects in animal models. Estrogen receptor beta (ER-β), for example, was found to co-localize in the hypothalamus with cells expressing oxytocin and vasopressin receptors in rodents (Alves et al., 1998). Female rats treated with exogenous sex hormones in the neonatal period showed higher levels of oxytocin receptor binding in the brain (Uhl-Bronner et al., 2005). Castrated hamsters had lower levels of AVPR1a receptor in their brains, unless they were treated with testosterone replacement (Delville et al., 1996b). In an animal model using ovariectomised rats, the HPA axis showed elevations in stress hormones when rats were restrained; this effect was buffered by administration of oxytocin only in the presence of exogenous estradiol replacement (Ochedalski et al., 2007).

Sex steroids have also been shown to be important to the behavioral effects of these neuropeptides. For example, knockout of either the oxytocin gene, or estrogen receptors α or β led to deficient social abilities in mice (Choleris et al., 2003). Central administration of vasopressin triggered aggression in rats, but the effect was lessened if they had been castrated, thereby lowering testosterone levels (Korte et al., 1990). The relevance of these findings to research looking at behavioral effects in humans remains unclear; a single intranasal dose of oxytocin in humans led to slight augmentation in peripheral testosterone levels, but no change in progesterone or estradiol levels (Gossen et al., 2012). The relationship between sex hormones and neuropeptides is supported, yet further complicated by literature showing that single doses of estradiol and testosterone administered in humans can lead to behavioral effects on social functioning and threat/reward perception that overlap with effects of oxytocin or vasopressin administration (Bos et al., 2012). While autism is significantly more common in males than females, there is no clear understanding of whether sex hormones, oxytocin, or vasopressin contribute to this difference, although various hypotheses have been proposed (Baron-Cohen et al., 2011).

### Dopamine

Dopamine is thought to contribute to the effects of oxytocin and vasopressin on social processes, potentially via its impact on the reward pathway. Specifically, research in prairie voles has highlighted the importance of dopamine in facilitating the partner preference formation via oxytocin and vasopressin manipulation. For example, dopamine receptor 2 (D2) blockade using various agents including haloperidol blocked partner preference behavior in prairie voles, while D2 agonists facilitated partner preference formation (Wang et al., 1999). This mechanism appeared to be mediated via dopaminergic activity in the nucleus accumbens, as evidenced by increased dopamine turnover in this area, and specificity of the effects of D2 blockade injected into this region in particular (Gingrich et al., 2000; Aragona et al., 2003). Activation of both the D2 receptor and oxytocin receptor was necessary to facilitate partner preference formation in voles; blockade of either receptor eliminated partner selection (Liu and Wang, 2003). Similarly, artificial up regulation of AVPR1a in voles using a viral vector led to increased partner preference formation, an effect that was blocked by D2 antagonism (Lim et al., 2004). Oxytocin neurons in the hypothalamic nuclei in rats have also been shown to express dopamine receptors (Baskerville et al., 2009).

Dopamine activity in the nucleus accumbens is central to behavioral reinforcement, reward, and motivation. Dopamine neurons which originate the ventral tegmental area (VTA) project to the medial prefrontal cortex, amygdala, and nucleus accumbens, while glutamatergic neurons in the medial prefrontral cortex project back to the nucleus accumbens and serve a regulatory function. Glutamate antagonists or oxytocin injected into the VTA in rodents decreased dopamine release in the frontal cortex, while increasing dopamine release in the nucleus accumbens, suggesting differential inhibitory regulation within this system (Takahata and Moghaddam, 2000; Melis et al., 2007). This regulation of dopamine in the nucleus accumbens by the ventral tegmental area via the prefrontal cortex in voles provides an example of a circuit driving social behavior. Inhibition of either GABA_A_ (gamma hydroxyl butyric acid) receptors or AMPA (α-amino-3-hydroxy-5-methyl-4-isoxazolepropionic acid) receptors in the ventral tegmental area also led to a decrease in dopamine activity in the prefrontal cortex, and an increase in dopamine in the nucleus accumbens, which was associated with increased partner preference formation (Curtis and Wang, 2005). Oxytocin, vasopressin and dopamine receptors are co-expressed in the medial prefrontal cortex in voles; higher D2 concentration and OXTR binding in this region is associated with greater monogamous behavior (Smeltzer et al., 2006). Simultaneously, variation in the density of OXTR in the nucleus accumbens of prairie voles (through viral mediated over-expression) accelerated partner preference formation (Ross et al., 2009). Along these lines, humans with ASD have been shown to have aberrant dopamine transporter distribution and function (Nakamura et al., 2010; Hamilton et al., 2013), while oxytocin administration in humans may be able to enhance the saliency of certain social cues (discussed further in subsequent sections).

In summary, oxytocin's effects on reward pathways, (including the nucleus accumbens, VTA, and prefrontal cortex) likely modulate the saliency of social stimuli. Given that oxytocin receptors have not been detected in the nucleus accumbens in human and primate brains, other areas may indirectly mediate this effect. Notably, the basal forebrain which stains densely with oxytocin receptors in humans receives many inputs from the prefrontal cortex, VTA, and nucleus accumbens, and indeed projects to various cortical regions (Sarter et al., 2009). It would follow that pituitary neuropeptides may selectively modulate signaling within the basal forebrain and surrounding areas, contributing to signaling within classic reward pathways involving dopamine in humans.

### Interneurons

A separate body of research proposes that the neuropeptide effects occur specifically on fast spiking interneurons. Interneurons serve a local regulatory function within microcircuits by impacting on the firing of principal neurons. Principal neurons drive the dominant signals and outputs propagated to other brain regions (Freund and Buzsaki, 1996). There have been numerous and varied attempts to classify interneurons based on their structure and function. Generally speaking, interneurons exert inhibitory signals on principal neurons through GABA. Fast spiking neurons are classified as such due to their low threshold to quickly deliver inhibitory signals.

Recent research in hippocampal cells *in vitro* has shown that oxytocin receptor agonism effectively strengthens the signal to noise ratio via its impact on interneurons. When exposed to oxytocin, these fast-spiking interneurons in the hippocampus increased their inhibitory output, thereby lowering background firing (noise) in the principal cell circuits. Simultaneously, the balance of excitatory to inhibitory transmission within the circuit was altered, such that the strength and fidelity of firing within the principal cells was increased and made more efficient, effectively enhancing the coordinated signal in the overall network (Owen et al., 2013). In a mouse model *in vivo*, interneurons expressing the oxytocin receptors in the frontal cortex were shown to be involved in social and sexual behavior (Nakajima et al., 2014). A similar mechanism may also exist with respect to the actions of vasopressin. It has previously been shown that application of vasopressin to hippocampal cells can enhance neurotransmission, leading to long-term potentiation (Rong et al., 1993; Chepkova et al., 1995). In rats, application of AVP to hippocampal tissue sections increased the frequency of inhibitory signals; this process was shown to occur due to AVP binding to AVPR1a, which through a G-protein mediated cascade, increased the excitability of interneurons, leading to increased GABA release. At the same time, AVP had an excitatory effect on principal neurons (of pyramidal type) in this circuit (Ramanathan et al., 2012). OXTR and AVPR1a were also shown to mediate reciprocal inhibitory effects in different regions within the rat amygdala via GABA activity (Huber et al., 2005). Of note, networks of GABAergic interneurons have also be characterized in the basal forebrain in primates (Walker et al., 1989).

Recent literature using optogenetic methods further support an association between the inhibitory GABA system, oxytocin, vasopressin, and behavior. Specifically, Knobloch et al. used an adenovirus vector to insert light sensitive channelrhodopsin molecules into the oxytocin axons of rat brains. They demonstrated that hypothalamic neurons have axonic projections extending directly to the amygdala, among other locations. When endogenous oxytocin was released as triggered by blue light, there was a local increase in GABAergic interneuron signaling within the amygdala, which was associated with a reduction in freezing behavior in fear conditioned rats (Knobloch et al., 2012). Cortical and brainstem networks may also be impacted in this way; a recent optogenetic study showed that oxytocin secretion via hypothalamic projections to the piriform cortex was necessary for social learning around both salient and aversive stimuli (Choe et al., 2015), while oxytocin projections to brainstem autonomic nuclei mediated heart rate variability (Pinol et al., 2014).

An imbalance of excitatory/inhibitory signaling during critical periods of development is an appealing explanatory theory for autism (Yizhar et al., 2011). A potential mechanism of action of oxytocin on GABA transmission occurs via modulation of chloride channel activity. In fetal rats, oxytocin increased intracellular chloride concentration in GABA neurons, thereby reducing neuronal excitation; this process was thoughts to protect the neonate from anoxic injury (Tyzio et al., 2006, 2014). Accordingly, chloride importer antagonists are currently being investigated as a potential treatment for ASD (Lemonnier et al., 2012).

### Section summary

In summary, data derived from animal studies have begun to tease apart a complex social network involving multiple brain structures potentially impacted by pituitary neuropeptides. Specifically, dopamine and serotonin appear to be important to encoding social information, potentially via their impact on reward pathways and anxiety, while serotonin may be involved in peptide secretion. Sex hormones appear to impact on the density of peptide receptor expression from early life, while both oxytocin and vasopressin can modulate stress responses in the hypothalamic-pituitary (HPA) axis. Recent research suggests that oxytocin and vasopressin may mediate their effects by activating inhibitory interneurons across subcortical and potentially cortical networks, including reward pathways. This model provides an appealing example of how these molecules may impact diffuse brain regions to strengthen signal outputs or fine tune inhibitory control. Further research using more advanced methods like optogenetics is anticipated to clarify these networks.

## Functional neuroimaging

Functional neuroimaging studies investigating the neural correlates of social processing began to emerge in the late 1990s and early 2000s. Investigators used blood oxygen level dependent imaging (BOLD) imaging to quantify local brain regions with increased activity in response to various social stimuli. Together, this research provides evidence for a large-scale social network in the human brain spanning multiple regions, including the amygdala, prefrontal, and orbitofrontal cortex, the insula, temporoparietal junction, and fusiform gyrus (Stanley and Adolphs, 2013). In the following section, we review functional neuroimaging literature in response to acute peptide administration in humans. Note that most studies include fMRI scans performed approximately 1 h after intranasal application, with data analyzed primarily via a region of interest approach. Neuroimaging correlates of common genetic variation in oxytocin and vasopressin receptors are not included in this review, but have been summarized elsewhere (Zink and Meyer-Lindenberg, 2012).

### Oxytocin

**Figure 3:**
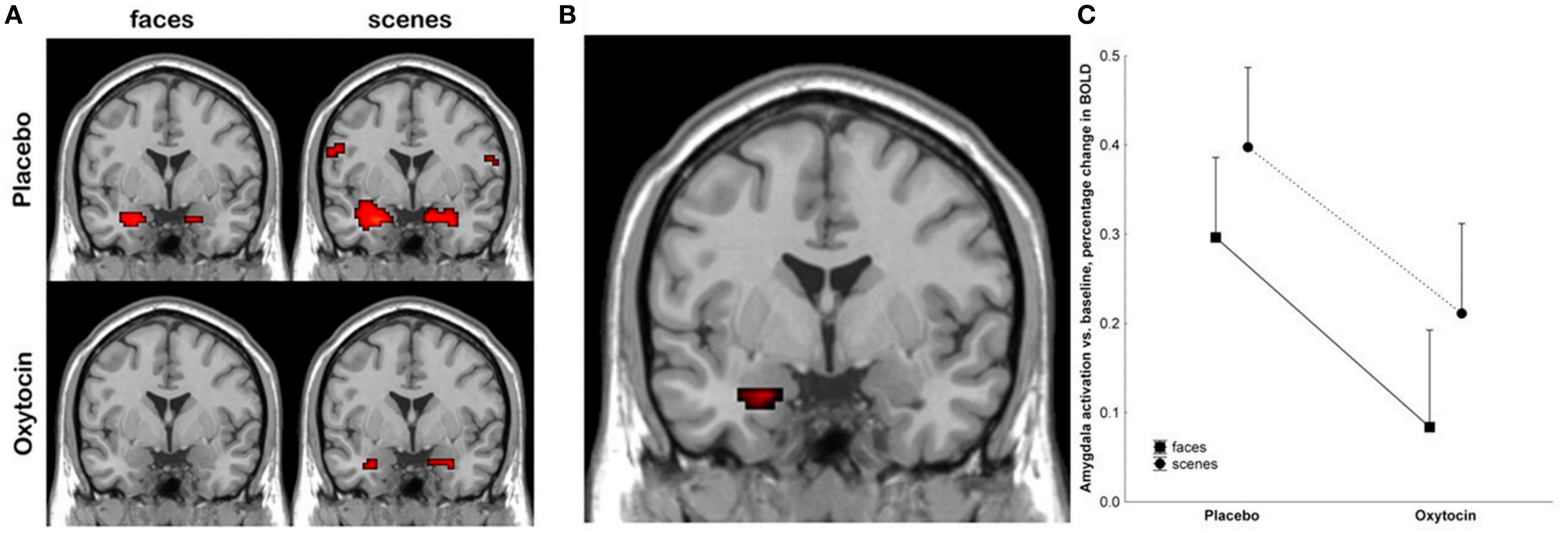
**Kirsch et al. (2005) showed that oxytocin attenuated amygdala activation in response to fearful stimuli**. **(A)** shows activation at the amygdala, with neural responses to fearful faces shown on the left, and to fearful scenes on the right, under placebo conditions (top), and after treatment with oxytocin (bottom). **(B)** shows the main effect of the drug in the left amygdala, where the signal was strongest. **(C)** plots BOLD levels at the amygdala using a region of interest analysis. Reproduced with permission from *J. Neurosci.* (Kirsch et al., 2005).

Kirsch et al. (2005) were one of the first groups to employ functional neuroimaging technology in order to try to better understand the mechanism by which oxytocin exerts behavioral effects in humans following acute administration. They administered intranasal oxytocin to 15 male subjects, and then had them watch fear inducing stimuli, including fearful faces, in an fMRI scanner. Using a region of interest approach focused on the amygdala, they found that oxytocin significantly reduced amygdala activation, and also reduced coupling between the amygdala and brainstem regions involved in autonomic arousal (see Figure 3) (Kirsch et al., 2005). The authors proposed that oxytocin attenuated the fear response at the level of the amygdala.

Domes et al. (2007) subsequently showed that this attenuated activity in the amygdala from oxytocin persisted in response to viewing other facial expressions also (e.g., happy, angry) (Domes et al., 2007). On exploratory whole brain analysis, they also identified reduced activation in many other brain regions including areas of the temporal lobe, thalamus, and frontal lobe. Subsequent investigators have showed a similar pattern of attenuated fMRI activity in the amygdala when treated with oxytocin during games of trust (Baumgartner et al., 2008), and in response to the sound of an infant crying (Riem et al., 2011) (see Table 2). Further studies suggested that the pattern of amygdala activation might vary within specific subsection of the amygdala, or depending on the valence of the emotional cue. For example, more than one study has shown that oxytocin increased amygdala activation in response to positive social information (Gamer et al., 2010; Rilling et al., 2012). However, there is significant variability within the functional neuroimaging literature, with bidirectional, and at times conflicting findings regarding amygdala activation in response to social cues (see Table 2). A recent meta-analysis of data suggested left insular hyperactivation emerges most consistently (Wigton et al., 2015).

**Table 2:** **fMRI changes in response to acute administration of oxytocin or vasopressin**.

**Study**	**Subjects**	**Task Description**	**Task Valence**	**Amygdala**	**Temporal lobe**	**Insula**	**Anterior cingulate**	**Prefrontal**	**Other**
**OXYTOCIN**									
Kirsch et al., 2005	15 M	Fearful stimuli, including face showing fear	Negative	↓L					↓Coupling between amygdala and brainstem
Domes et al., 2007	13 M	Looking at fearful, angry, happy faces	Negative	↓R	↓*L*			↓*L*	↓*L cerebellum, medulla, thalamus, R pre- and L post-central gyrus (negative valence), ↓L paracentral gyrus (negative and positive valence)*
			Positive	↓R	↓				
Baumgartner et al., 2008	49 M	Financial game involving trust	Neutral	↓		↓L			↓Brainstem L (midbrain), ↓caudate, ↓L post-central gyrus
Petrovic et al., 2008	27 M	Neutral faces previously paired with negative experiences	Negative	↓R	↓		↓R	↓vmPFC ↓vlPFC ↓L OFC	↓R fusiform face area
Singer et al., 2008 (AH)	20 M	Observing pain inflicted on another	Negative						No difference on social/ empathy tasks
Gamer et al., 2010	46 M	Classifying fearful and happy faces	Negative	↓L (ant)					↑Superior colliculus, and connectivity of superior colliculus and posterior amygdala
			Positive	↑L (ant)					
Domes et al., 2010	16 F	Rating fearful, happy, angry faces	Negative	↑L	↑L	↑L		↑	↑R brainstem (fear), ↑ fusiform gyrus,
			Positive		↑L	↑L			
Riem et al., 2011	42 F	Sound of infant crying	Negative	↓R		↑		↑	
Rilling et al., 2012	60 M	Prisoners dilemma game (Reciprocated or un-reciprocated cooperation)	Negative					↑L	↑L caudate (positive) ↑Amygdala -insula and -temporal connectivity, ↓Amygdala-brainstem connectivity
				Positive	↑L					
Lischke et al., 2012	14 F	Viewing threatening and non-threatening scenes	Negative	↑	↑*L*				↓*R SMA for positive stimuli*
Wittfoth-Schardt et al., 2012	21 M	Viewing pictures of child faces (own vs. other)	Positive						↓Activity and functional connectivity in globus pallidus, ↓ *L hippocampus*
Striepens et al., 2012	70 M	Aversive social stimuli	Negative	↓R		↑L (recall)			↑Coupling between amygdala, insula and PFC
Groppe et al., 2013	28 F	Social incentive delay task (reward vs. punishment anticipation)	Positive		↑*L*				↑ VTA activity, ↑*L occipital,*
			Negative						↑ VTA, ↓*precentral gyrus, ↓L cuneus, ↓R post-central gyrus,*
			Both			↑			↑VTA, *↑L thalamus, striatum, brainstem, ↑R thalamus,*
Sripada et al., 2013	15 M	Resting state	N/A					↑mPFC	↑Amygdala connectivity to mPFC and ACC
Riem et al., 2013	42 F	Resting state while recalling love withdrawal	Negative						↑Connectivity between posterior cingulate cortex and brainstem
Rilling et al., 2014	87 F	Prisoners dilemma game (Reciprocated and un-reciprocated cooperation)	Negative						Note lack of findings as previously seen in male cohort on same task/ protocol (Rilling et al., 2012)
			Positive	↓L					
Voorthuis et al., 2014	50 F	Emotion recognition in infant faces	Both		↑L			↑L IFG	
Riem et al., 2014	50 F	Reading the mind in the eyes task	Both		↑L	↑L			
Feng et al., 2014	153 M 151 F	Prisoners dilemma game (reciprocated cooperation)	Positive					↑M ↓F	↑M ↓F caudate/putamen
Kanat et al., 2015b	49 M	Detection of angry, happy, or neutral expression	Negative	↓					↓*Fusiform, brainstem and striate cortex* ↓*connectivity amygdala and fusiform (for angry and fearful stimuli)*
			Positive	↓				↓	
Kumar et al., 2014	15 M	Resting state	N/A						↓Connectivity between amygdala and precuneus with OT
Eckstein et al., 2015	62 M	Conditioned fear response followed by extinction	Negative	↓				↑	↑*Connectivity from PFC to precuneus, and precuneus to amygdala*
Kanat et al., 2015a	50 M	Fearful faces, and eyes only (asked to assess gender)	Negative	↓R	↓*L*		↓*L*		↓R pulvinar (trend)
Chen et al., 2015	153 M 151 F	Prisoners dilemma game (unreciprocated cooperation)	Negative	↓ M		↓ M			
**OXYTOCIN IN PARTICIPANTS WITH ASD**									
Gordon et al., 2013	17 ASD	Reading the mind in the eyes task vs. vehicle classification task	Both		↑L				↑R precentral gyrus, ↑ striatum and nucleus accumbens, ↑ cerebellum and pons, ↑Posterior cingulate, precuneus, ↑L parahippocampal region, ↑ L inferior parietal lobule,
Domes et al., 2013	28 M 14 ASD	Face matching and house matching task	Neutral	↑R (in ASD)					
Domes et al., 2014	28 M 14 ASD	Face emotion recognition task	Both	↑L (ASD)	↑*R*				↑*IFG, SMA, cerebellum, and superior parietal lobe*
Watanabe et al., 2014	40 M ASD	Making decisions about social information	Both	↓			↑	↑mPFC	↑Connectivity from mPFC to ACC
Aoki et al., 2014	20 M ASD	Sally-Anne task (inferring emotions)	Both			↑ R (ant)			
**VASOPRESSIN**									
Zink et al., 2010	20 M	Face matching task	Negative					↓mPFC	↓Subgenual cingulate region ↓Connectivity in this area
Zink et al., 2011	20 M	Face matching task (familiar vs. unfamiliar)	Negative		↓TPJ				
Rilling et al., 2012	60 M	Prisoners dilemma game (Reciprocated and un-reciprocated cooperation)	Both						↑BNST, lateral septum and stria terminalis. ↑Amygdala insula connectivity
Brunnlieb et al., 2013	42 M	Black and white drawings of social situations without facial information	Negative	↑R					Increased functional connectivity between amygdala and mPFC
			Positive						
Feng et al., 2014	153 M 151 F	Prisoners dilemma game (reciprocated cooperation)	Positive			↑M ↓F			↑R SMG M ↓R SMG F
Chen et al., 2015	153 M 151 F	Prisoners dilemma game (unreciprocated cooperation)	Negative	↓M		↓M			

Table Includes studies with tasks related to social processing, or resting state, in control subjects or those with ASD. fMRI, functional magnetic resonance imaging. Boxes for regions left blank were either not studied or indicated in the respective manuscript, or differences were non-significant. Findings in italics are from secondary/exploratory analyses. Task valence refers to the emotional quality (e.g., positive = happy; negative = fearful) of the task in which the fMRI findings are described. L, left side only; R, right side only; M, male subjects; F, female subjects; N/A, not applicable; ↑, increased activity; ↓, decreased activity; vlPFC, ventrolateral prefrontal cortex; vmPFC, ventromedial prefrontal cortex; mPFC, medial prefrontal cortex; IFG, inferior frontal gyrus; Ant, anterior; VTA, ventral tegmental area; ASD, autism spectrum disorder; SMG, supramarginal gyrus; SMA, supplementary motor area; TPJ, temporoparietal junction; BNST, basal nucleus of stratum terminale; ACC, anterior cingulate cortex. Most studies used intranasal oxytocin administration, of 16–24 IU, or intranasal vasopressin administration of 20–40 IU.

One potential explanation for observed differences across studies is the seemingly differential effects of oxytocin administration depending on the sex of the participant. Prior to 2010, most fMRI studies of oxytocin recruited only male participants. Domes et al. (2010) were the first to investigate oxytocin's effects on face processing in a group of females only; they detected a pattern somewhat opposite to that observed in males, with increased amygdala activity while observing negative facial expression in response to oxytocin treatment (Domes et al., 2010). This effect persisted despite controlling for estradiol and progesterone levels. Increased amygdala activation in females in response to negative or threatening social information was replicated in a small sample (Lischke et al., 2012), although subsequent studies did not find the same effect (see Table 2).

Rilling and colleagues have published several manuscripts attempting to tease apart the sex effects of this response, with increasingly large sample sizes, using the prisoner's dilemma game. In this task, participants must choose whether to risk cooperating to achieve the best outcome for both participants, or defect against their partner and achieve a positive outcome for themselves only (Declerck et al., 2014). In males, treatment with either AVP or oxytocin increased brain activation in the basal forebrain, amygdala, hippocampus and striatum, and treatment with AVP increased cooperative behavior in the game (Rilling et al., 2012). In women, however, neither peptide led to activation in these brain regions; oxytocin instead decreased amygdala activity (Rilling et al., 2014). Plasma estrogen levels did not modulate this effect. A subsequent paper confirmed differential sex effects in the same, but larger group of participants; findings were more specific, however, with increased activity in the frontal pole, medial prefrontal cortex, and caudate/putamen in men, but decreased or no activity in these regions in women, in response to reciprocal cooperation while being treated with oxytocin (Feng et al., 2014). The amygdala was no longer implicated with the larger sample size. Other investigators recently showed that women who scored lower on a social perception task (reading the mind in the eyes), performed better in response to oxytocin administration, an effect that was associated with enhanced activation in the superior temporal gyrus and insula (Riem et al., 2014). The ventral tegmental area showed increased activation in response to oxytocin administration in another group of women (Groppe et al., 2013).

Subsequent studies have attempted to tease apart the differential fMRI findings in response to oxytocin administration by looking at brain connectivity specifically (for a more detailed review Bethlehem et al., 2013). Some have found, for example, that oxytocin can both reduce amygdala activation in response to negative stimuli (Striepens et al., 2012), but also increase connectivity of the amygdala to other regions including the insula, and prefrontal cortex, and anterior cingulate, potentially facilitating memory of social information (Striepens et al., 2012; Sripada et al., 2013). Resting state MRI data showed increased connectivity between the posterior cingulate and brainstem in response to oxytocin treatment (Riem et al., 2013). Others have shown that oxytocin reduced connectivity between the amygdala and precuneus (Kumar et al., 2014).

### Oxytocin in ASD

Recent data have used fMRI technology to attempt to understand the potential effects of oxytocin administration in individuals with ASD. In a small pilot study, Domes et al., showed that participants with ASD had lower activity as compared to controls in the right amygdala, fusiform gyrus, and occipital region during face processing, and that intranasal oxytocin administration increased the right amygdala activity in the affected group (Domes et al., 2013). The same investigators subsequently showed that oxytocin improved emotion recognition abilities in adults with ASD, and that this effect correlated with increased left amygdala activation on fMRI. Note that under placebo conditions, amygdala reactivity was comparable in both the ASD and control group in this sample (Domes et al., 2014). In another study in adults with ASD, oxytocin increased the otherwise decreased brain activation in the medial prefrontal cortex (Watanabe et al., 2014). Increased activity in the right anterior insula in response to oxytocin treatment coincided with increased accuracy in inferring others' emotions (Aoki et al., 2014). Gordon et al., also found that oxytocin enhanced fMRI activity in regions including the amygdala, nucleus accumbens, and orbitofrontal cortex during social tasks in a group of children with ASD (Gordon et al., 2013).

### AVP

Far fewer studies have been conducted looking at AVP administration in humans on fMRI. One of the first trials found no effects of AVP administration on amygdala activity, although there was a corresponding decrease in medial prefrontal cortex hyperactivity in the treatment group as compared to placebo (Zink et al., 2010). A subsequent paper by the same group also showed that a region which showed increased activity in the temporoparietal junction in response to unfamiliar faces, was no longer hyperactive in participants treated with AVP (Zink et al., 2011). Notably, behavioral and symptom surveys did not identify any neuropsychiatric effects of AVP administration in these studies. Rilling et al. compared fMRI signals and functional connectivity measures in male participants during the prisoner's dilemma game, following administration of either oxytocin or vasopressin. Interestingly, both peptides led to behavioral changes, with more cooperative behavior. Oxytocin increased caudate activation in response to positive cooperation and also increased left amygdala activity on whole brain analysis. AVP increased activity in the bed nucleus of the stria terminalis and lateral septum. Both peptides reduced amygdala connectivity to the brainstem (Rilling et al., 2012). Subsequent studies also found sex effects in response to AVP administration; bilateral insula, and right supramarginal gyrus activity was increased in men, while decreased in women, during reciprocated cooperation (Feng et al., 2014).

### Summary

Overall, functional neuroimaging literature following acute administration of oxytocin and vasopressin support their potential role in social information processing as evidence by neural activation in regions implicated in social brain networks. Findings in this regard are complicated by (1) significant heterogeneity in the tasks studied, (2) the potential of differential effects of these peptides depending on the sex of the participants and the valence of the emotional stimuli, and (3) the large number of studies of relatively small sample size. A few themes stand out overall: (1) Brain activation patterns in response to peptide administration span several different regions; the amygdala, prefrontal cortex, insula and temporal lobe emerge most frequently across studies. Some studies have also detected differential activation patterns in the basal forebrain and brainstem. (2) Many investigators have shown changes in functional connectivity between various structures in the above listed regions in response to peptide administration. (3) A small number of studies including participants with ASD suggest that aberrant functional activation patterns in response to social stimuli may be partially corrected following acute treatment with oxytocin. The implications of this information as it relates to previous sections are discussed in the next section.

## Discussion, synthesis, and implications in autism

In summary, oxytocin and vasopressin are neuropeptides synthesized in the hypothalamus and secreted into the peripheral vasculature through the posterior pituitary. In rodents, a functionally separate process also mediates central release of these peptides from hypothalamic neurons into central nervous systems; it is presumed that a similar mechanism is at play in humans as well. Centrally secreted neuropeptides are thought to diffuse through the extracellular fluid into surrounding tissue, where they exert their neuromodulatory effects; specific axons also deliver peptides directly to distant brain regions. The oxytocin and vasopressin receptors are G-protein linked, and activate various downstream pathways, which vary by cell type and organ system.

Limited literature on the distribution of these neuropeptide receptors in the central nervous system implicates the basal forebrain (including the nucleus basalis, and diagonal band), brainstem, and potentially the limbic system as areas of oxytocin binding in humans and primates, while AVP receptors appear more diffusely throughout the brain. The basal forebrain has previously been described as serving a regulatory function, consolidating various external inputs and amplifying signals in relevant downstream cortical targets (Givens and Sarter, 1997). It is functionally connected within a reward network involving the nucleus accumbens and ventral tegmental area (Sarter et al., 2009). Indeed, various neurotransmitter systems and brain regions that have afferent or efferent connections with the basal forebrain and brainstem regions have been implicated in studies investigating the mechanisms behind the neurobehavioral effects of these peptides. Blocking dopamine within the reward pathways described above can eliminate many of the behavioral effects of oxytocin in rodents. Recent literature demonstrating that oxytocin and vasopressin can increase the signal to noise ratio and enhance coordinated signaling via their activity on interneurons could tentatively link these concepts together. In essence, could these pituitary neuropeptides act on interneurons within the basal forebrain to consolidate and strengthen signaling to relevant downstream cortical and subcortical regions via cholinergic, glutamatergic, or monoamine neurotransmitter pathways in response to external social stimuli?

Functional neuroimaging can provide information on brain activation patterns. Earlier studies proposed that the neural effects of oxytocin and vasopressin occurred as a result of attenuated amygdala activity in response to fearful stimuli. Subsequent studied found differences in BOLD signaling more diffusely, although clustering within specific social brain areas (including the prefrontal cortex, insula, amygdala, and temporal lobe). Importantly, significant heterogeneity across studies highlight how the neural effects of oxytocin and vasopressin likely depend on the type of social stimulus, the sex of the participant, and other contextual factors. While oxytocin and vasopressin receptors have been detected in the insula, amygdala, and cerebral cortex in rodents, data are inconsistent in these regions in primates and humans. The neuroimaging literature would instead support a model in which peptide effects on subcortical networks subsequently impact social appraisals and downstream cortical activation patterns. Indeed, numerous studies highlight how the functional connectivity between the amygdala, brainstem, anterior cingulate, insula, temporal lobe, and prefrontal cortex is altered under the influence of these pituitary neuropeptides. Along these lines, the cholinergic basal forebrain has prominent connections to the cortex, as well as other subcortical and brainstem structures; it can also regulate amygdala activity (Power, 2004).

Tentatively, many associations can be drawn between the putative mechanisms of action of oxytocin and vasopressin and hypotheses regarding the pathophysiology of ASD. For example, numerous immune system differences have been detected in autism, while specific inflammatory cytokines have been shown to alter the expression of oxytocin receptors *in vitro*. Sex differences in the manifestation and incidence of autism have been well-described; at the same time, the oxytocin and vasopressin systems have been shown to interact with sex hormones in numerous ways. Imbalances in excitatory/inhibitory neurotransmission have increasingly been characterized in neurodevelopmental disorders such as ASD, while cellular physiology research suggests that both oxytocin and vasopressin can alter this balance by acting on interneurons. Aberrant functional and structural connectivity in ASD has been detected using various neuroimaging modalities and both oxytocin and vasopressin may be able to alter connectivity within brain social networks. Although at present, data do not appear to suggest that disruption of the oxytocin or vasopressin systems necessarily contributes to the etiology of ASD (e.g., only a single case of a rare variant disrupting OXTR has been described), these molecules do seem to impact on social functioning, presenting a potential therapeutic target.

In the context of advancing technology, important next steps for this field include determining more precisely the anatomic location of CNS receptor expression via radiolabeled ligands, in typical individuals, and importantly, in those with ASD and other neurodevelopmental disorders. While rodent models permit elegant proof of concept experiments, results must be confirmed and replicated in primates, if not humans. For example, a recent neuroimaging paper in macaques provided evidence of functional overlap with humans in brain activation patterns in response to oxytocin (Liu et al., 2015). Quantification of the level of inter individual variation in receptor expression will be important as well. Characterization of the three dimensional and long-range patterns of receptor expression within nervous tissue in humans may prove informative, and may be possible with novel techniques (Chung et al., 2013). Functional connectivity analyses focusing on brainstem and forebrain regions, at different and more proximal time point following peptide administration may help to better characterize the sequence of changes taking place in the CNS. Investigations to support or refute an interneuron mediated increase in the signal to noise ratio in specific networks in humans in response to peptide administration will prove interesting and informative to the field. Excitingly, emerging research efforts are underway hoping to harness the therapeutic potential of these molecules with respect to treating social deficits in neurodevelopmental disorders. Given that peptide penetrance of the blood brain barrier has proven to be a challenge, exploration of other compounds that act on peptide receptors may also prove beneficial. Additionally, it is hoped that clinical biomarkers within this system (e.g., common genetic receptor subtypes) may be able to predict variable subgroup responses, in order to optimize the therapeutic potential of these peptides.

## Author contributions

DB authored the manuscript. EA developed the research topic, provided guidance, editing and supervision.

## Funding

This research was supported by the grant IDS-11-02 from the Ontario Brain Institute.

### Conflict of interest statement

Danielle A. Baribeau has no financial conflicts to disclose. Evdokia Anagnostou has consulted to Roche. She has received grant funding from Sanofi-Aventis Canada and SynapDx.
